# Supplementary material for: Thermally boosted upconversion and downshifting luminescence in Sc2(MoO4)3:Yb/Er with two-dimensional negative thermal expansion
Source: Nat Commun. 2022 Apr 19;13:2090. doi: 10.1038/s41467-022-29784-6 (PMC9019035; doi:10.1038/s41467-022-29784-6)
Supplement: Supplementary file 1 — Supplementary information [file 41467_2022_29784_MOESM1_ESM.pdf]

Supplementary Information

Thermally boosted upconversion and downshifting luminescence in $Sc_2(MoO_4)_3$:Yb/Er with two-dimensional negative thermal expansion

Jinsheng Liao[1, +, *], Minghua Wang[1, +], Fulin Lin[2, 3, +], Zhuo Han[1, +], Biao Fu[1], Datao Tu[2, *], Xueyuan Chen[2, *], Bao Qiu[4, *], He-Rui Wen[1]

[1]School of Chemistry and Chemical Engineering/Jiangxi Provincial Key Laboratory of Functional Molecular Materials Chemistry, Jiangxi University of Science and Technology, Ganzhou, Jiangxi, 341000,P.R. China.

[2]CAS Key Laboratory of Design and Assembly of Functional Nanostructures, and Fujian Key Laboratory of Nanomaterials, Fujian Institute of Research on the Structure of Matter, Chinese Academy of Sciences, Fuzhou, Fujian 350002, China

[3]Xiamen Institute of Rare Earth Materials, Haixi Institute, Chinese Academy of Sciences, Xiamen 361021, China

[4]Ningbo Institute of Materials Technology & Engineering (NIMTE), Chinese Academy of Sciences, Ningbo, Zhejiang, 315201, P. R. China

[*] Corresponding author

E-mail address: jsliao1209@126.com, dttu@fjirsm.ac.cn, xchen@fjirsm.ac.cn, qiubao@nimte.ac.cn

[+]These authors contributed equally to this work.

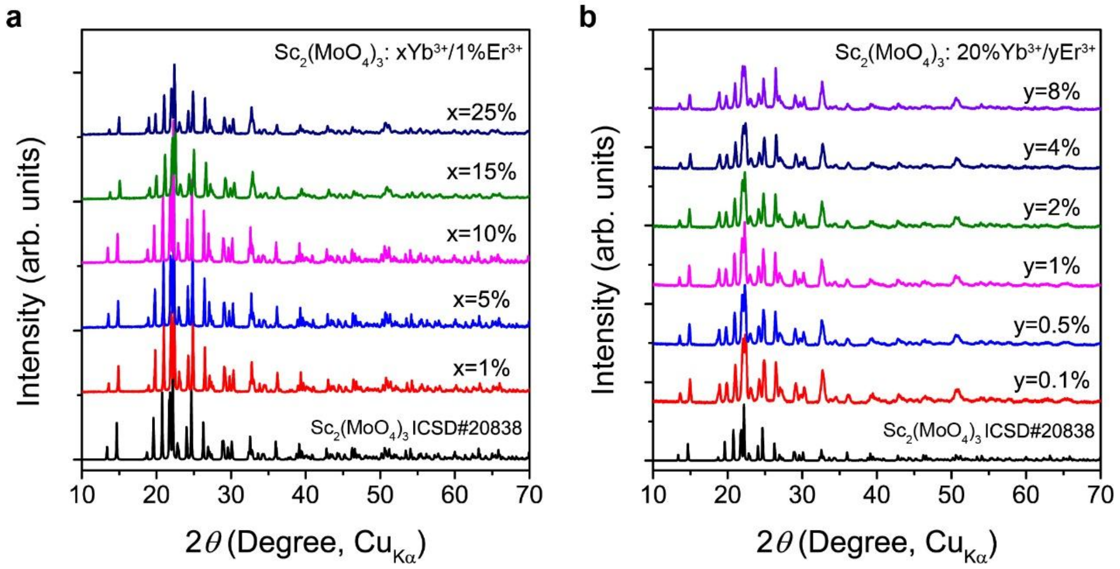

Supplementary Figure 1| Structural characterization. a. XRD patterns of $Sc_2(MoO_4)_3$:xYb/1%Er phosphors with different Yb^{3+} concentrations at room temperature. **b.** XRD patterns of $Sc_2(MoO_4)_3$:20%Yb/yEr phosphors with different Er^{3+} concentrations at room temperature. All the structures of the as-prepared samples are well consistent with the orthorhombic $Sc_2(MoO_4)_3$ (*ICSD#20838*) without any observable impurities.

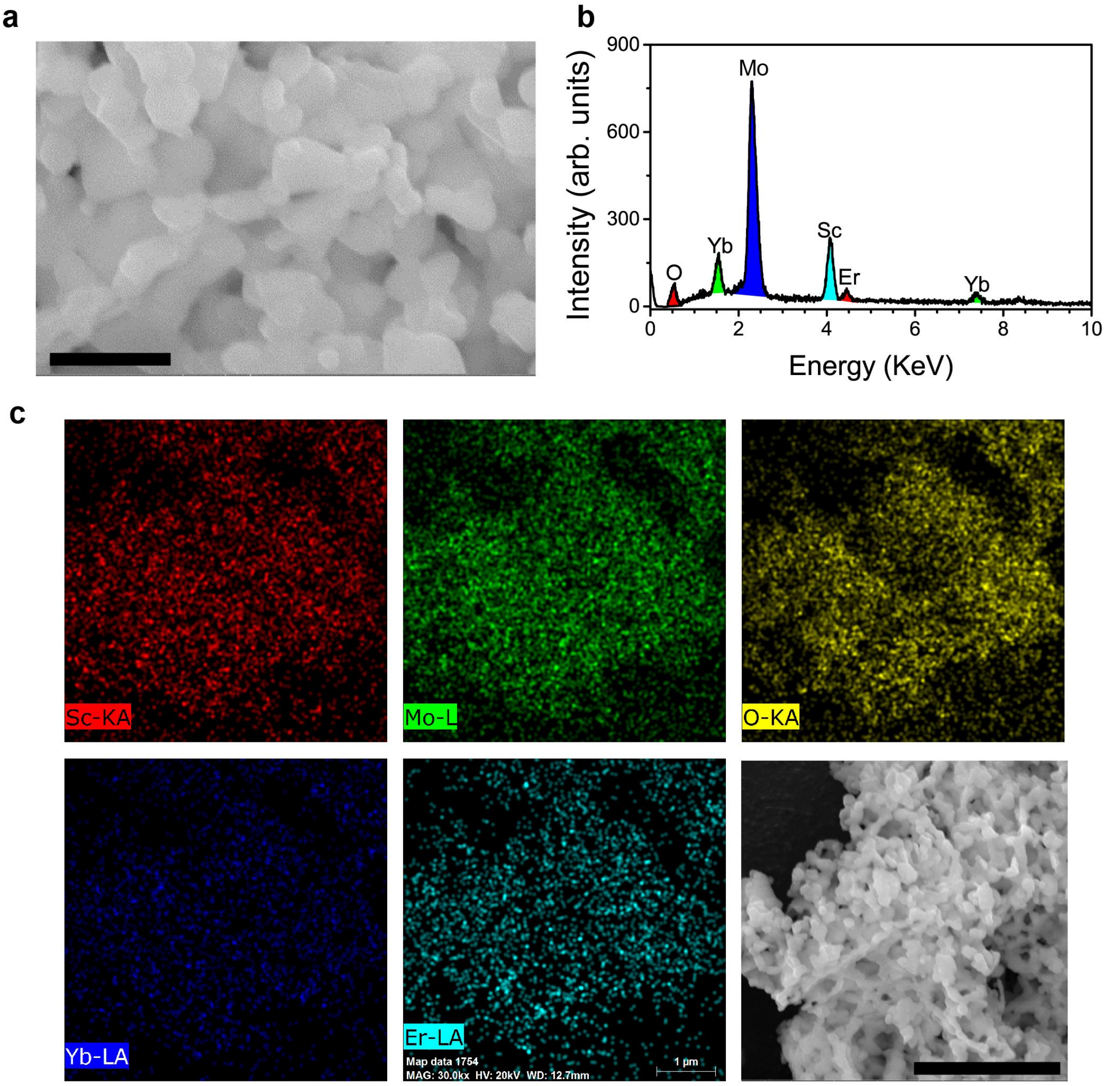

Supplementary Figure 2| Morphology and chemical compositions. a. Typical SEM images of the $Sc_2(MoO_4)_3$:20%Yb/1%Er phosphors. The scale bar is 1 μm. **b.** EDS data taken from a single particle. **c.** Elemental mappings of Sc, Mo, O, Yb and Er in $Sc_2(MoO_4)_3$:20%Yb/1%Er particle. The scale bar is 5 μm. It can be observed that the as-prepared $Sc_2(MoO_4)_3$:Yb/Er samples are microcrystals with sizes of 1-2 μm and Sc, Yb, Er, Mo and O elements are homogeneously distributed in the matrix.

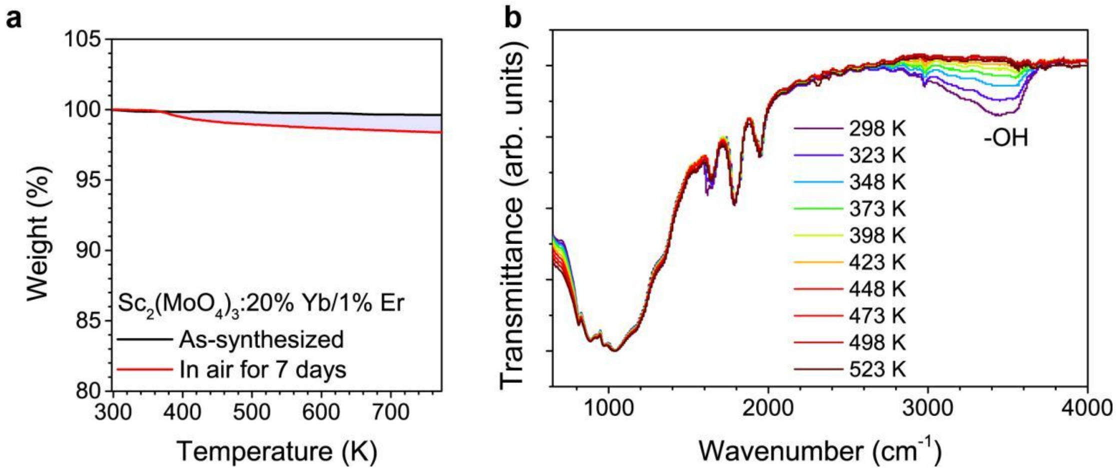

Supplementary Figure 3| Water molecules analysis. a. Thermogravimetry curves of the $Sc_2(MoO_4)_3$:20%Yb/1%Er phosphor. A weight loss of about 1% for $Sc_2(MoO_4)_3$:20%Yb^{3+}/1%Er^{3+} was detected by heating from 298 to 420 K, which may result from the removal of water molecules. **b.** Temperature-dependent Fourier transform infrared spectra of $Sc_2(MoO_4)_3$:20%Yb/1%Er phosphor. The typical absorbance peak at 3400-3500 cm^{-1}, corresponding to the asymmetric vibration of -OH was markedly suppressed with the temperature from 298 to 420 K, which further verified the existence and removal of water molecules in the $Sc_2(MoO_4)_3$:20%Yb/1%Er phosphor.

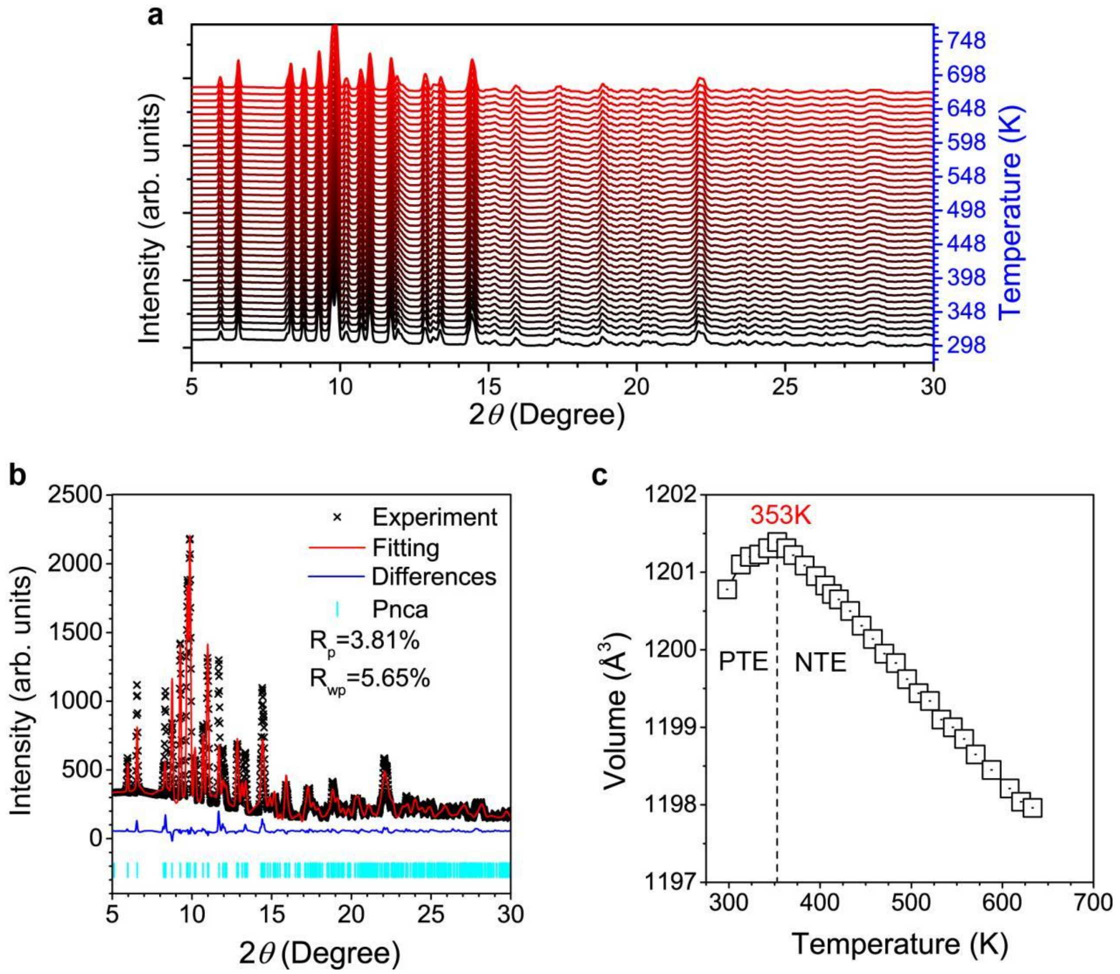

Supplementary Figure 4| Structural characterization. a. Temperature-dependent in situ *SXRD* patterns. **b.** Rietveld refinement of SXRD for the typical $Sc_2(MoO_4)_3$:Yb/Er sample measured at room temperature. **c.** Temperature-dependent changes of the unit cell volumes. For the temperature range of 298-353 K, the $Sc_2(MoO_4)_3$:Yb/Er phosphors exhibit positive-thermal expansion due to the existence of water molecules in the microchannels. At higher temperatures above 353 K, the $Sc_2(MoO_4)_3$:Yb/Er phosphors exhibit typical negative-thermal expansion properties of $Sc_2Mo_3O_{12}$ compounds.

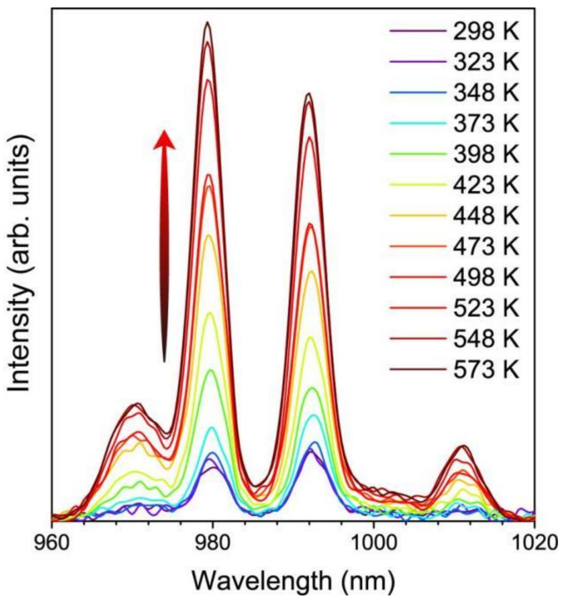

Supplementary Figure 5| Spectroscopic characterization. Temperature-dependent excitation spectra of the $Sc_2(MoO_4)_3$:20%Yb/1%Er phosphors by monitoring the emission of Er^{3+} at 1538 nm. The intensity of the excitation peak increased with the temperature rising from 298 to 573 K.

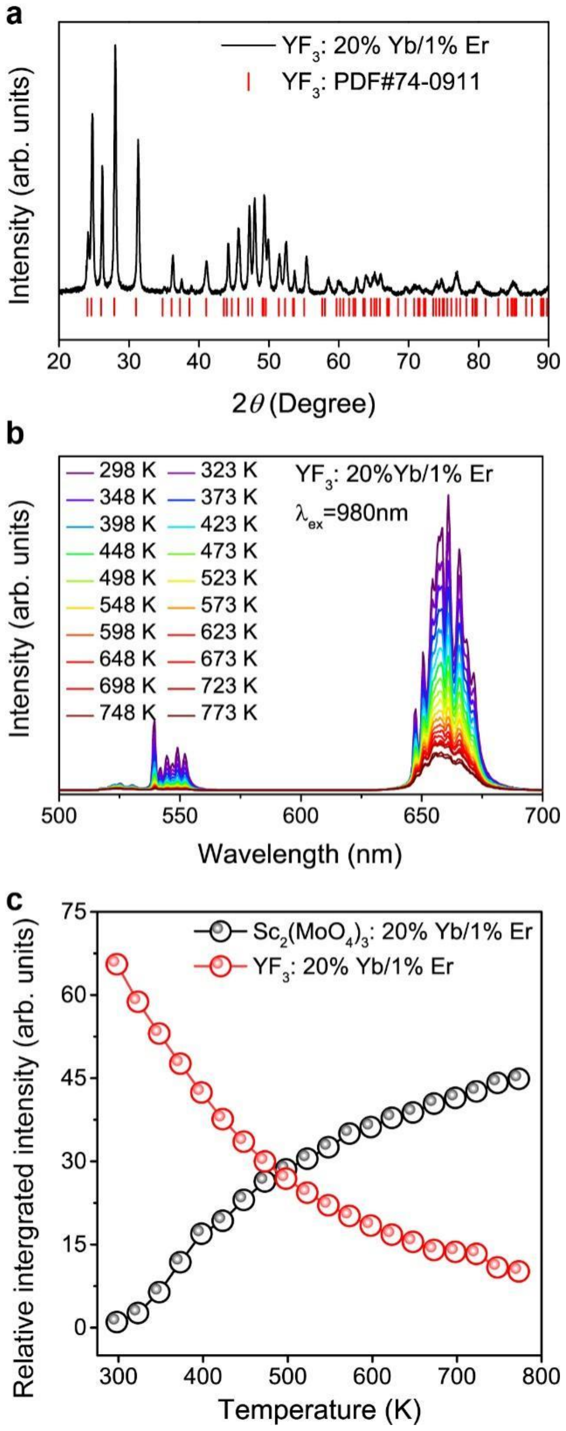

Supplementary Figure 6| Spectroscopic characterization. a. XRD patterns of YF_3:20%Yb/1%Er phosphor. **b**. Upconversion emission spectra of the YF_3:20%Yb/1%Er as a function of temperature under 980 nm excitation. **c**. Comparison of the relative integrated intensity of the upconversion emission of the YF_3:20%Yb/1%Er and $Sc_2(MoO_4)_3$:20%Yb/1%Er phosphor, wherein the data of $Sc_2(MoO_4)_3$:20%Yb/1%Er originate from Fig. 3b. The overall upconversion

luminescence intensity of YF_3:20%Yb/1%Er is much higher than that of $Sc_2(MoO_4)_3$:20%Yb/1%Er at 298 K. Nevertheless, the integrated upconversion intensity of $Sc_2(MoO_4)_3$:20%Yb/1%Er phosphor is 4.5 times higher than that of YF_3:20%Yb/1%Er counterpart at 773 K.

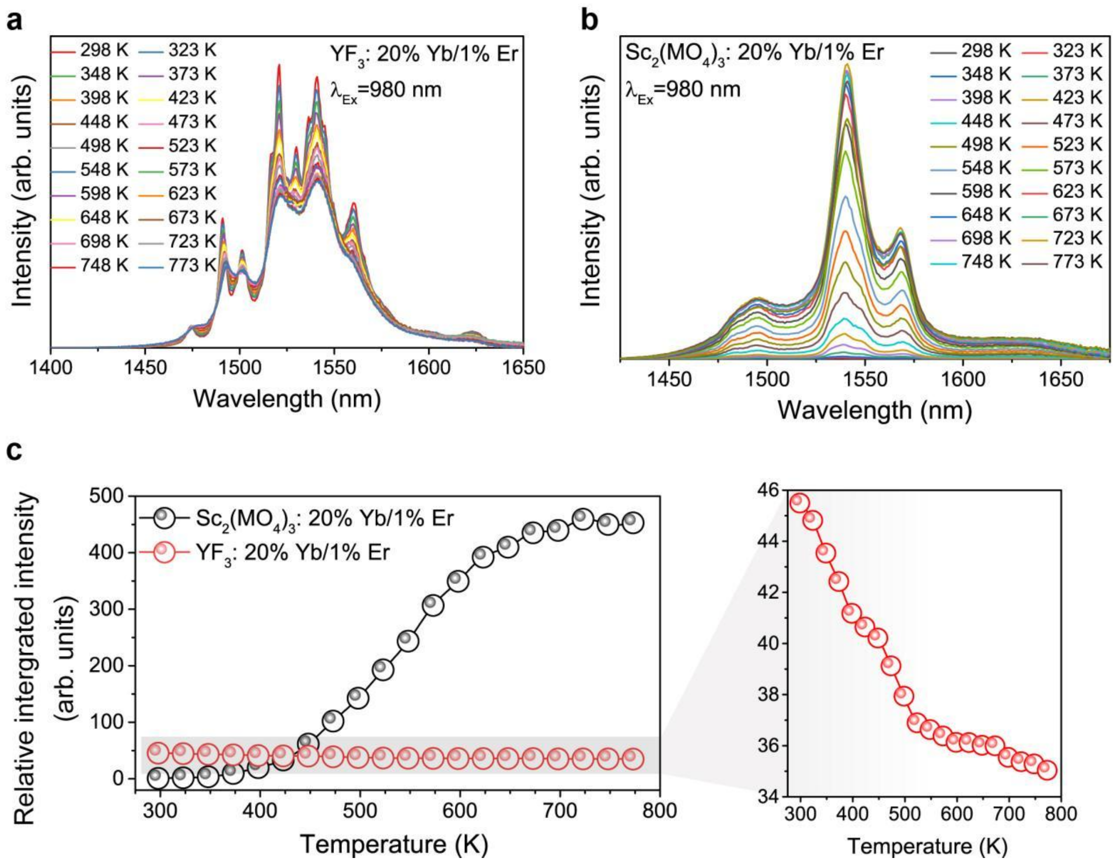

Supplementary Figure 7| Spectroscopic characterization. a. and **b.** Downshifting emission spectra of the YF_3:20%Yb/1%Er and $Sc_2(MoO_4)_3$:20%Yb/1%Er phosphor as a function of temperature under 980-nm excitation. **c.** Comparison of the relative integrated intensity of the downshifting emission spectra of the YF_3:20%Yb/1%Er and $Sc_2(MoO_4)_3$:20%Yb/1%Er phosphor. The enlarged data of YF_3:20%Yb/1%Er are displayed on the right side. The overall downshifting luminescence intensity of YF_3:20%Yb/1%Er is much higher than that of $Sc_2(MoO_4)_3$:20%Yb/1%Er at 298 K. Nevertheless, the integrated downshifting luminescence intensity of $Sc_2(MoO_4)_3$:20%Yb/1%Er phosphor is 12.9 times higher than that of YF_3:20%Yb/1%Er counterpart at 773 K.

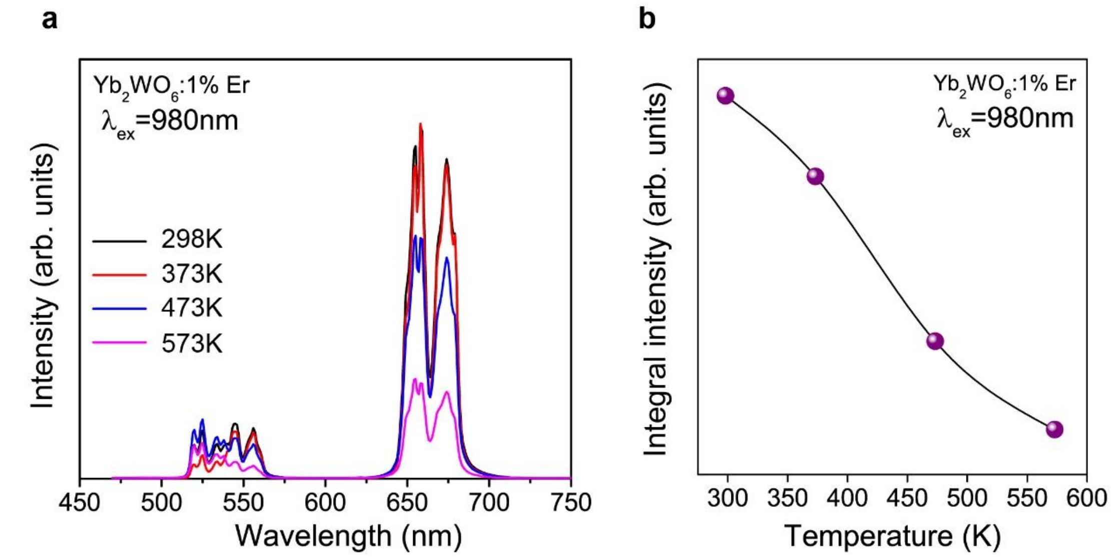

Supplementary Figure 8| Upconversion emission spectra of Yb_2WO_6:1%Er^{3+} phosphor. a. Upconversion emission spectra of the Yb_2WO_6:1%Er^{3+} phosphors as a function of temperature under 980-nm excitation. **b.** Integral emission intensity of Yb_2WO_6: 1%Er^{3+} as a function of temperature. Thermally quenched luminescent emission was detected when the temperature increased from 298 to 573 K, since the increased distance between Yb^{3+} to Er^{3+} would reduce the ET efficiency from Yb^{3+} to Er^{3+}.

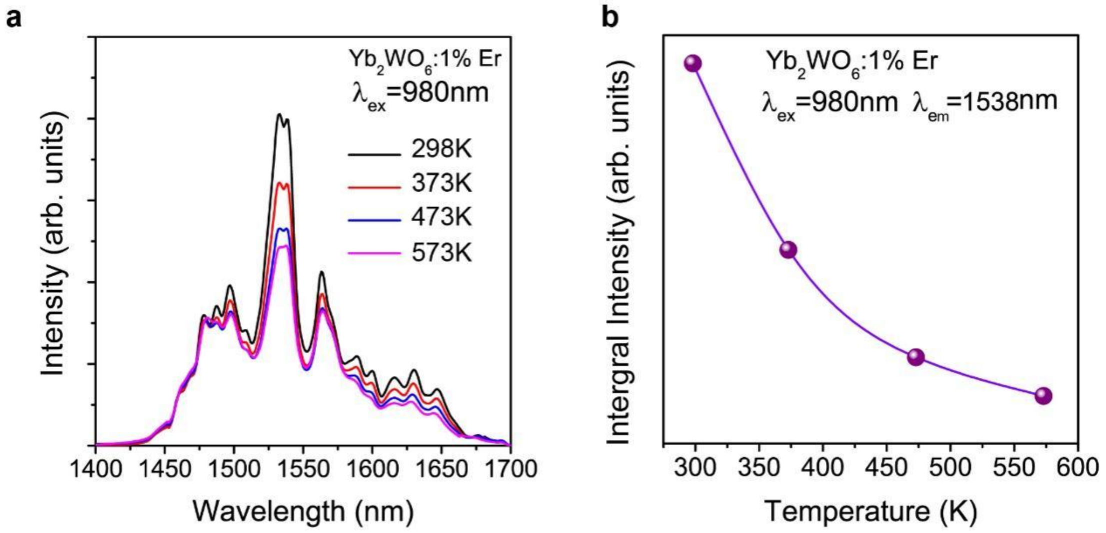

Supplementary Figure 9| Downshifting emission spectra of Yb_2WO_6:1%Er phosphor. a. Downshifting emission spectra of Yb_2WO_6:1%Er phosphors. **b.** Integral emission intensity of Yb_2WO_6:1%Er phosphors as a function of temperature. Thermally quenched luminescent emission was detected when the temperature increased from 298 to 573 K, since the increased distance between Yb^{3+} to Er^{3+} would reduce the ET efficiency from Yb^{3+} to Er^{3+}.

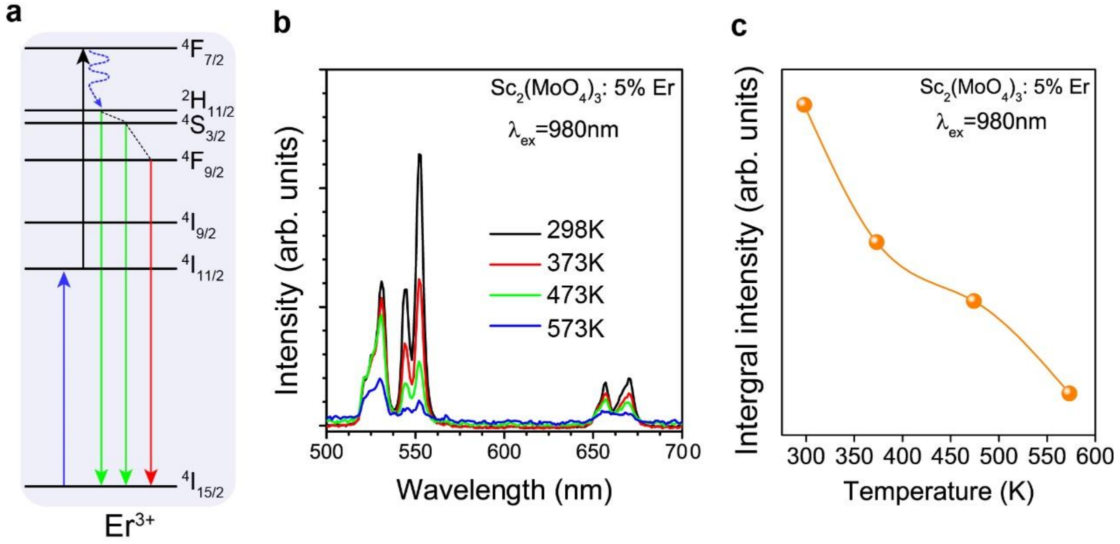

Supplementary Figure 10| Upconversion emission spectra of $Sc_2(MoO_4)_3$:5%Er^{3+} phosphor. a. Simplified energy level diagram showing upconversion process via excited-state absorption of Er^{3+}. **b.** Upconversion emission spectra of the $Sc_2(MoO_4)_3$:5%Er^{3+} phosphors as a function of temperature under 980 nm excitation. **c.** Integral emission intensity of $Sc_2(MoO_4)_3$:5%Er^{3+} phosphors as a function of temperature. The emission intensity of Er^{3+} decreased with the increase of temperature upon 980-nm excitation without the ET from Yb^{3+} to Er^{3+} due to the detrimental cross-relaxation process between Er^{3+} ions.

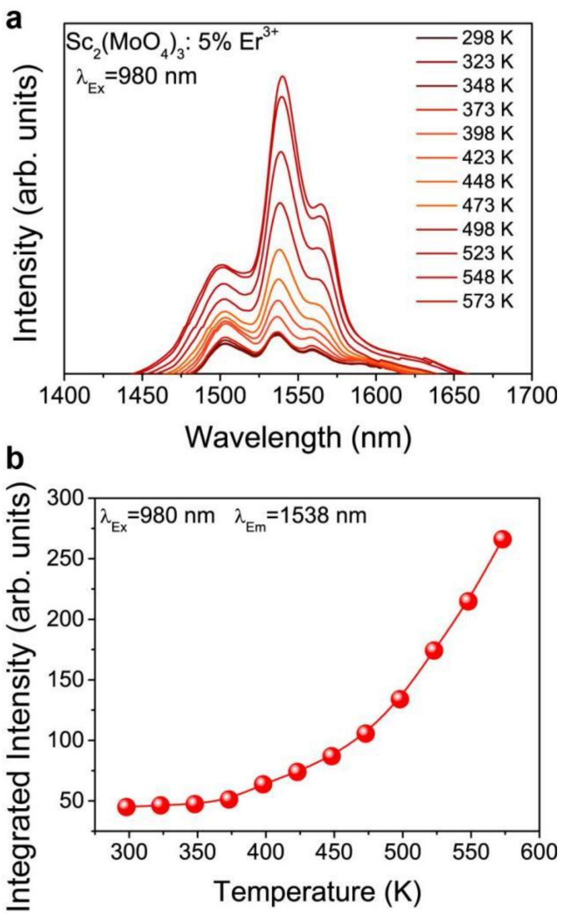

Supplementary Figure 11| Downshifting emission spectra of $Sc_2(MoO_4)_3$:5%Er phosphor. a. Downshifting emission spectra of $Sc_2(MoO_4)_3$:5%Er as a function of temperature under 980-nm excitation. **b** Integrated NIR emission intensities of $Sc_2(MoO_4)_3$:5%Er^{3+} as a function of temperature. The integrated emission of Er^{3+} was improved by 5.9 fold with the increase of temperature from 298 to 573 K.

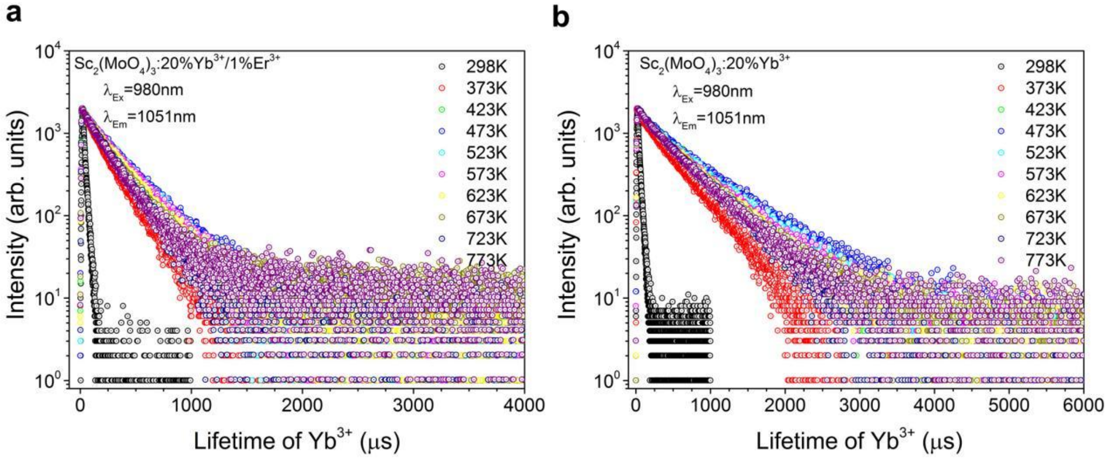

Supplementary Figure 12| Temperature-dependent luminescence decay curves of Yb^{3+}. a. and **b.** Temperature-dependent luminescence decay curves of $^2F_{5/2}$ excited state of Yb^{3+} in Yb^{3+}/Er^{3+}-codoped and Yb^{3+}-doped $Sc_2(MoO_4)_3$, respectively. Without the energy transfer from Yb^{3+} to Er^{3+}, the excited state ($^2F_{5/2}$) lifetime of Yb^{3+} in $Sc_2(MoO_4)_3$:Yb phosphors was determined to be longer than that of $Sc_2(MoO_4)_3$:20%Yb/1%Er at the same temperature. Moreover, it can be determined that the energy-transfer efficiency from Yb^{3+} to Er^{3+} in $Sc_2(MoO_4)_3$:20%Yb/1%Er increased gradually when the temperature was raised from 298 to 473 K.

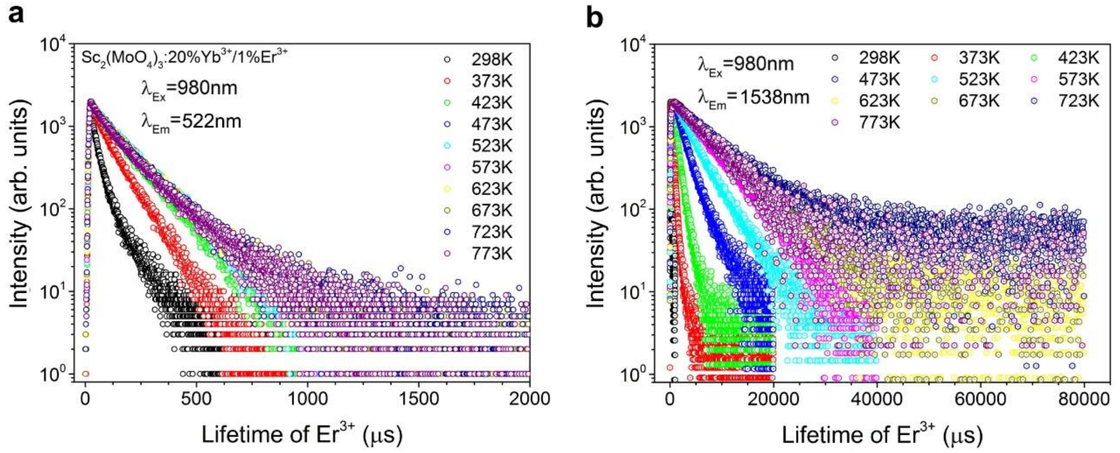

Supplementary Figure 13| Temperature-dependent luminescence decay curves of Er^{3+}. a. and **b.** Temperature-dependent luminescence decay curves of $^2H_{11/2}$ and $^4I_{13/2}$ excited states of Er^{3+} in Yb^{3+}/Er^{3+}-codoped $Sc_2(MoO_4)_3$, respectively. The photoluminescence lifetime of $^2H_{11/2}$ (522 nm) of Er^{3+} increased from 29.77 to 142.36 μs with the temperature from 298 to 473 K, and remained essentially stable above 473 K. For $^4I_{13/2}$ (1538 nm) of Er^{3+}, the photoluminescence lifetime increased markedly from 61.2 μs to 7789 μs as the temperature raised from 298 to 698 K, and decreased as the higher temperature above 698 K.

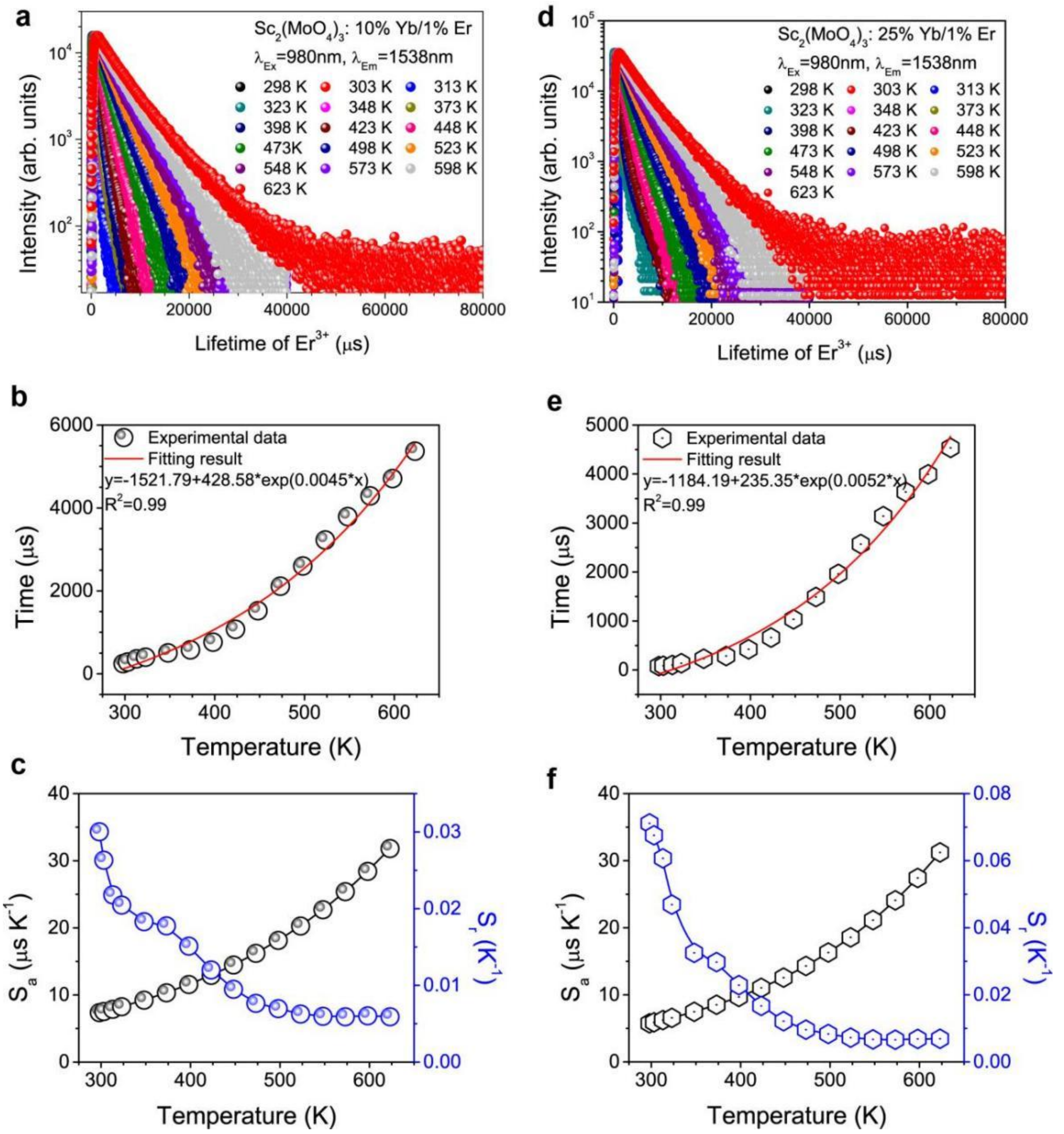

Supplementary Figure 14| Lifetime-based luminescence thermometry based on $Sc_2(MoO_4)_3$:Yb/Er phosphor. a. Temperature-dependent luminescence decay curves of $^4I_{13/2}$ excited states of Er^{3+} in $Sc_2(MoO_4)_3$:10%Yb^{3+}/1%Er^{3+}. **b**. Experimentally measured and exponentially fitted plots of lifetime τ of $Sc_2(MoO_4)_3$:10%Yb/1%Er at different temperatures. **c**. Calculated absolute sensitivity S_a and relative sensitivity S_r versus temperature based on the $Sc_2(MoO_4)_3$:10%Yb/1%Er. **d**. Temperature-dependent luminescence decay curves of $^4I_{13/2}$ excited states of Er^{3+} in $Sc_2(MoO_4)_3$:25%Yb^{3+}/1%Er^{3+}. **e**. Experimentally measured and exponentially fitted plots of lifetime τ of $Sc_2(MoO_4)_3$:25%Yb/1%Er at different temperatures. **f.** Calculated absolute sensitivity S_a and relative sensitivity S_r versus temperature based on the $Sc_2(MoO_4)_3$:25%Yb/1%Er.

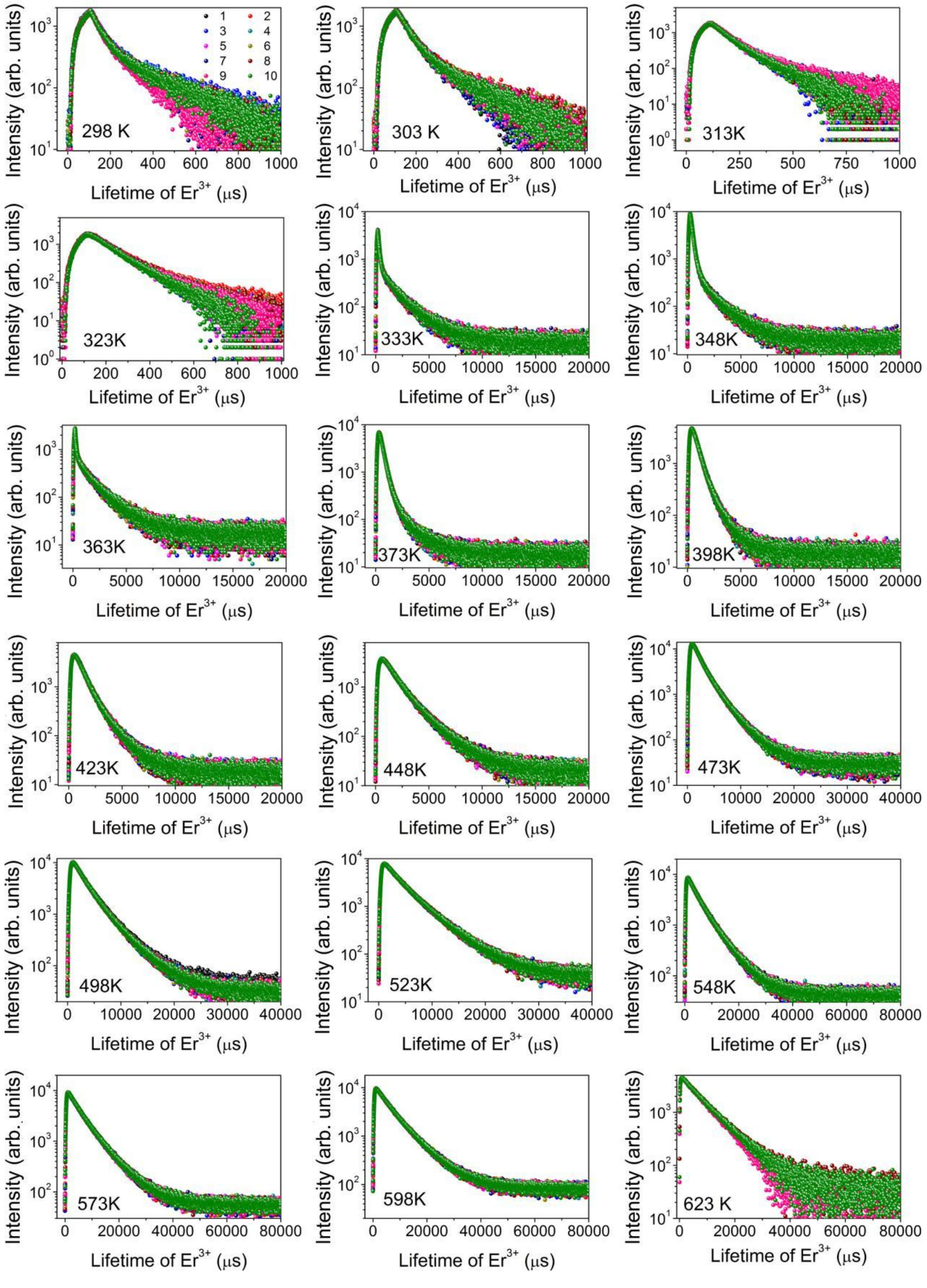

Supplementary Figure 15| Temperature-dependent luminescence decay curves of Er^{3+}. Temperature-dependent luminescence decay curves of $^4I_{13/2}$ excited states of Er^{3+} in $Sc_2(MoO_4)_3$:20%Yb/1%Er at different temperatures from 298 to 623 K. Each temperature point was measured by ten consecutive measurements.

Supplementary Table 1|. Lifetime-based luminescence thermometry parameters of xYb/1%Er-codoped $Sc_2(MoO_4)_3$ with different Yb^{3+} concentrations. Accordingly, $Sc_2(MoO_4)_3$:20%Yb/1%Er phosphors exhibited the optimal S_a of 53.0 μsK^{-1} and S_r of 12.3% K^{-1}.

Sample	S_a (μs/K)	S_r (%/K)
$Sc_2(MoO_4)_3$:10%Yb/1%Er	31.8	3.0
$Sc_2(MoO_4)_3$:20%Yb/1%Er	53.0	12.3
$Sc_2(MoO_4)_3$:25%Yb/1%Er	31.2	7.1
